# Health-related quality of life in inflammatory bowel disease: a comparison of patients receiving nurse-led versus conventional follow-up care

**DOI:** 10.1186/s12913-022-08985-1

**Published:** 2022-12-31

**Authors:** Line Alvestad, Lars-Petter Jelsness-Jørgensen, Rasmus Goll, Anne Clancy, Thomas Gressnes, Per Christian Valle, Ann Ragnhild Broderstad

**Affiliations:** 1 UiT, The Arctic University of Norway, Tromsø, Norway; 2 University Hospital of North Norway, UNN Harstad, Tromsø, Norway; 3 Østfold University College, Fredrikstad, Norway; 4 Department of Gastroenterology, Østfold Hospital Trust, Kalnes, Norway; 5 UiT, The Arctic University of Norway, Tromsø, Norway

**Keywords:** Inflammatory bowel disease, IBD nurse specialist, Health-related quality of life, Micro-team, Multidisciplinary team, Quality of Life, Quality of care, Inflammatory Bowel Disease Questionnaire

## Abstract

**Background:**

Inflammatory bowel disease (IBD), consisting of Crohn's disease (CD) and ulcerative colitis (UC), is a chronic disorder with a considerable negative impact on health-related quality of life (HRQoL). During the past decade, IBD nurse specialists have been increasingly involved in follow-up care of IBD outpatients, in a consultative and coordinating role, closely cooperating with gastroenterologists.

Whether patients’ HRQoL differs between nurses’ follow-up care (NF) and conventional follow-up care (CF) has not been widely researched and the aim of this study was to compare two different follow-up regimes with respect to patients’ HRQoL.

**Methods:**

This cross-sectional, multicenter study involved seven centers; five organized as CF, two as NF.

**Results:**

A total of 304 patients aged 18–80 years, 174 females and 130 males, were included, of whom 140 received care under the NF model and 164 under the CF model. Participants in the NF group had a statistically significant higher median total score on the Inflammatory Bowel Disease Questionnaire (IBDQ) (*p*-value < .001). This pattern could also be seen in the sub-scores of the different IBDQ domains. Despite a trend of higher IBDQ score in all domains in the NF model, the overall result in our study did not reach the limit of 16 points, defined as clinically significant. A higher proportion of NF patients had IBDQ scores defined as remission, as well as a statistically significant higher frequency of outpatient check-ups during a two-year follow-up period.

**Conclusions:**

Nurse-led models are not inferior to conventional models with regards to patient reported HRQoL except in the social domain where the model showed to be clinically significant better. Further studies are needed to advance efforts to implement these models and increase access to IBD care.

## Introduction

Inflammatory bowel diseases (IBD), comprising mainly of Crohn’s disease (CD) and ulcerative colitis (UC), are lifelong, fluctuating disorders with the potential to cause long-term morbidity and a negative impact on patients’ health-related quality of life (HRQoL) [1–3]. Due to the early onset of IBD as well as a variety of symptoms, IBD patients often need frequent contact with the health care system [4].

As a direct consequence of IBD, patients often have challenges and needs that exceed the limits of a traditional clinical consultation, in which the main focus is on disease control and treatment. The traditional follow-up service comprising physician-dominated dialogues has been criticized by many patients for its lack of patient-centeredness, focus on HRQoL and comprehensive information [5–7]. Many patients with IBD experience fears and concerns as well as unmet needs [8]. However, limited time and resources are barriers preventing physicians from meeting these requirements [8].

During the last two decades, various IBD health care models have been suggested, including multidisciplinary teams and “micro teams” (MT). Multidisciplinary teams, often used in teaching hospitals, have been established and designed to manage complex cases by sharing collective experience and expertise [9]. These IBD teams should at least include gastroenterologists, an IBD nurse, surgeons, radiologists and stoma nurse [10]. The MT comprises small working groups of doctors, nurses and auxiliaries [11–13]. Hence an IBD MT consists of an IBD nurse specialist in close collaboration with a gastroenterologist and other relevant specialists, and is applicable to all hospitals regardless of size and complexity. The MT is designed to run the ordinary care of IBD patients, but hold the possibility to expand the team when necessary. In this form of organization, the nurse can provide rapid and direct access to care that includes social, physical and psychological support [14]. The European Crohn’s and Colitis Organisation underlines the need for continuity of care and proper access to outpatient clinics, including IBD nurse specialists [15]. Moreover, an IBD nurse can have a pivotal and autonomous role, providing more comprehensive consultations, ensuring care coordination, planning and evaluating treatment [12].

Even though the IBD nursing role is generally accepted as beneficial to the patient, it is not well defined [12]. To date there is no nationally or internationally established consensus on the level of education for IBD nursing, but the role is frequently held by an experienced IBD nurse, working autonomously at an advanced level in cooperation with gastroenterologists and other specialists [14]. Each national or local hospital can stipulate requirements for IBD nurses, along with its own expectations and objectives for this role [12]. Furthermore, the number of studies concerning different follow-up models in IBD is still limited and there is a need to investigate the role of nurses in improving quality of life outcomes [14]. Hence, the main question addressed in this cross-sectional study was to compare HRQoL in patients attending either an IBD nurse model as part of a micro team or conventional follow-up (NF vs. CF). Our hypothesis was that the IBD nurse model was non-inferior to conventional follow-up. Therefore, the aim of the study was to compare the models by measuring HRQoL.

## Materials and methods

### Study design, population and recruitment

This cross-sectional, multicenter study recruited participants from seven outpatient clinics in northern and southern Norway from June 2016 to June 2017. The NF model had been established at two of the seven clinics. The total number of IBD patients attending these different hospitals were identified from the hospitals` electronic medical records, and the diagnoses were based on the International Classification of Diseases (ICD-10) diagnosis codes K50 and K51 [16].

The study included patients aged between 18 to 80 years, with at least two years disease duration. The latter was based on the requirements of other endpoints investigated in the current project. Patients with stoma were excluded from participation.

Invitation letters, including study information and questionnaires, were sent to potential participants who then filled out the consent form and questionnaires and returned them in a prepaid envelope to the principal investigator. The invitations were sent separately from the IBD follow-up appointments. Non-responders received one reminder.

### Data collection

Socio-demographic characteristics including sex, age, smoking habits, educational level and current medication were collected. Clinical data including diagnosis, date of diagnosis, disease classification and IBD-medication were accessed from the patients’ medical records by the principal investigator in cooperation with one of the gastroenterologists in the study group. Patients had originally been diagnosed by a gastroenterologist based on clinical, endoscopic, histological and radiological findings. During our review of the medical records of the study patients, we re-evaluated all cases using the Montreal classification, to eliminate misdiagnoses as far as possible. This included the classification of disease location and behavior in CD and the disease extent in UC [17].

Additionally, we recorded the number of outpatient consultations from inclusion time and two years back in time.

#### Assessment of HRQoL

HRQoL was investigated using the Inflammatory Bowel Disease Questionnaire (IBDQ), a disease-specific and a widely used instrument designed to measure the effects of IBD on daily functioning and quality of life during the two weeks prior to filling in the questionnaire [18–21]. The Norwegian version (N-IBDQ) has been translated and validated and contains 32 items divided into five different domains: Bowel Function I (7 items), Bowel Function II (5 items) Emotional Function I (11 items), Emotional Function II (5 items) and Social Function (4 items) [22]. Responses are graded on a seven-point Likert scale from one (a very severe problem) to seven (not a problem). The IBDQ gives a possible score range of 32 to 224, where a higher score indicates better HRQoL [21, 23]. A difference of 16 points is regarded as clinically significant [21, 24]. IBDQ scores ≥ 180 are associated with remission and patients with scores ≤ 130 are associated with severely active disease [24]. Missing values were handled in accordance with McMaster`s recommendations, and was imputed as follows: no response for a particular question within a domain of the IBDQ was given the mean score for the other items of the sub-score. If two or more responses within a domain were lacking, the domain was encoded as “missing”. If more than four responses were missing for the entire IBDQ, the IBDQ was not scored and was excluded from further analysis.

### The IBD nurse follow-up care model

The NF models examined in this study were organized in MT with a flexible patient-centered proactive approach. The MT included IBD nurses, gastroenterologists and if necessary other specialists, e.g. surgeons, stoma nurse, nutritionist, rheumatologists, dermatologists or ophthalmologists. The IBD nurse conducted independent consultations at the IBD outpatient clinic and all patients were offered a minimum of one consultation per year, mostly more frequently. The appointments included clinical and medical assessments, as well as a focus on the physical and psychosocial aspects of the disease. If required, a gastroenterologist was consulted. Patients on biological therapy consulted the IBD nurse approximately three times a year, and the gastroenterologist at least once a year. All patients had access to a nurse-led direct telephone line and could be given an appointment at short notice. The NF MT models had been in operation for 10–15 years. The IBD nurses involved had different levels of further education and clinical experience from IBD follow-up care that ranged from two to 15 years.

In those of the Norwegian hospitals that have organized IBD care as MT, the medical follow-up care is a responsibility delegated to nurses by the physician in charge. IBD nurses are obliged to report any issues of concern that fall outside their scope of practice like assessment of serious illness and the need for surgery. The nurses are not allowed to prescript medication or referrals to other specialists and the gastroenterologist have to countersign the medical reports made by the nurses.

### Conventional follow-up model

The CF model was based on traditional medical appointments at outpatient clinics. In the CF, patient follow-up care was provided by a gastroenterologist, internist or a resident physician. The five control centers differed in the number of gastroenterologists, and the patient often met different physicians during the follow-up period. One center had several gastroenterologists in short-term temporary positions, while the other centers had one or more gastroenterologists in a permanent position.

### Statistical analysis

Descriptive statistics and frequencies were used to characterize the study population. Results are reported as medians, percentages and mean scores with standard deviation. A chi-square test was used to explore the relationship between categorical variables. To detect differences between continuous variables, the non-parametric Mann-Whitney U (MWU) test and the Student’s t-test were performed. When using the MWU-test, comparison of NF and CF required adjustment to correct for over- or under-representation of sociodemographic and clinical characteristics. The relevant variables weighted to correct for these differences were respectively; age, age categories, disease duration, Aminosalicylic acid (5-ASA) and Biologics. Statistical significance was set at *p < 0.05*. All tests were two-sided, using a 95% confidence interval. All statistics were performed with IBM SPSS Statistics, software version 25.0.

## Results

**Fig. 1:**
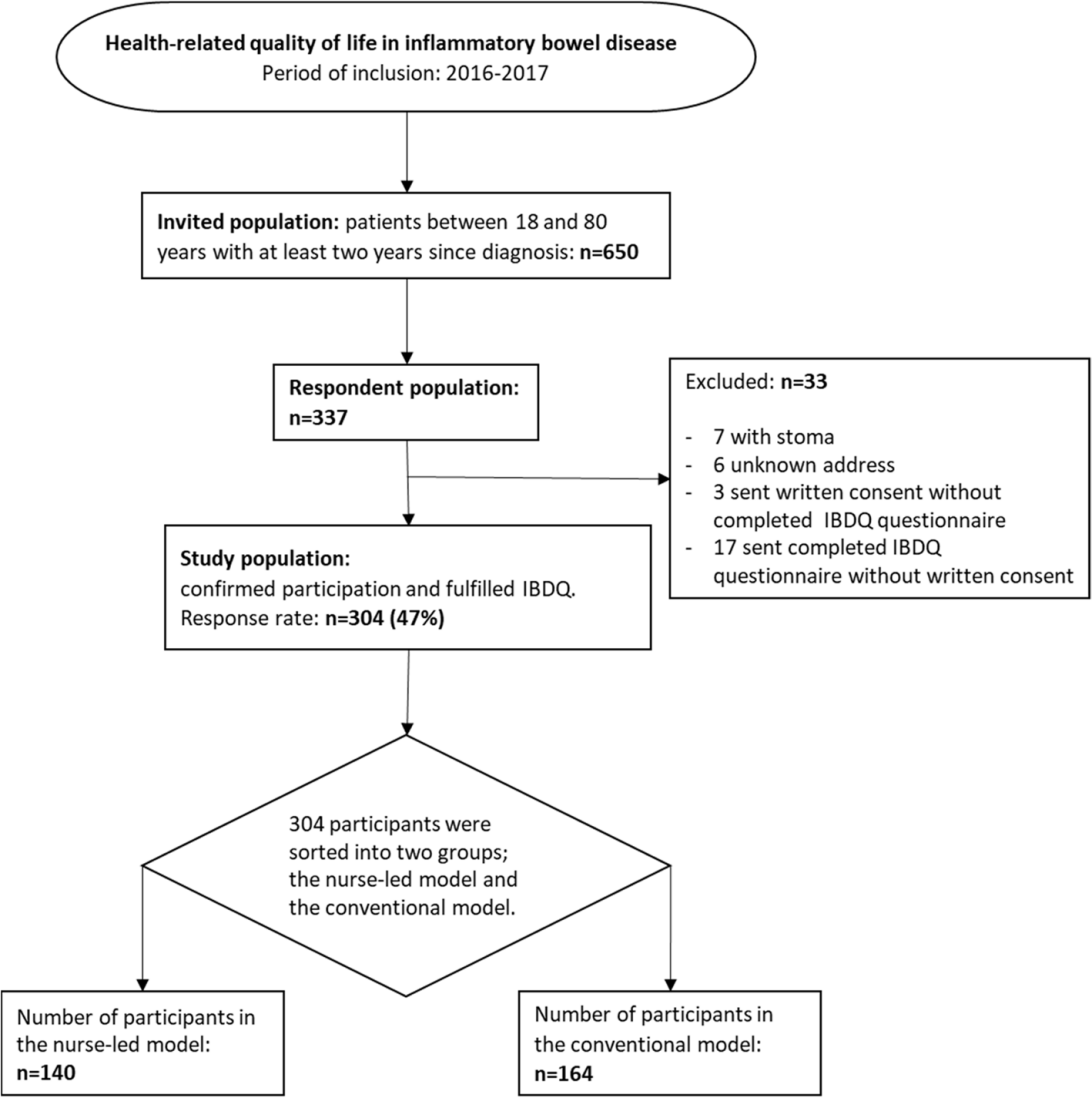
Flowchart of patients included in the study

A total of 304 patients (47% of the 650 invited) were included, of whom 140 received care under the NF model and 164 under the CF model. The inclusion process is presented in Fig. 1.

### Sociodemographic and clinical characteristics

The sociodemographic factors and clinical data are presented in Table 1. In brief, participants in the NF group had a statistically significant higher mean age and a longer disease duration than those in the CF group, while a higher proportion of CF patients were on biologics. Clinically, the disease classification was similar for both groups.

**Table 1:** Sociodemographic and clinical data by follow-up care models

	Nurse-led follow-up *n* = 140	Conventional follow-up *n* = 164	***P - value***
**Age, mean (SD)**	50 (12.9)	45 (14.5)	<.001(t-test)
**Age categories n (%)**			
18-39	31 (22)	58 (35)	0.012 (χ^2^)
40-80	109 (78)	106 (65)	
**Sex** n (%)			
Male	63 (45)	67 (41)	
Female	77 (55)	97 (59)	ns
**Current smoker** n (%)	50 (37)	61 (38)	ns
**Education level** n (%)			
Compulsory school only	12 (9)	22 (13)	
Upper secondary school	50 (36)	69 (42)	ns
Higher education < 4 years	34 (24)	42 (26)	
Higher education ≥ 4 years	44 (31)	31 (19)	
**Disease duration,** years mean (SD)	14.8 (10.1)	10.7 (8.2)	<.001 (t-test)
**IBD classification**^**a**^**n (%)**			
UC extent	*n* = 92 (59)	*n* = 65 (41)	
E1 ulcerative proctitis	20 (21)	6 (9)	ns
E2 left-sided UC	39 (42)	29 (45)	ns
E3 extensive UC	33 (36)	30 (46)	ns
CD localization	*n* = 48	*n* = 98	
L1 ileal	24 (50)	43 (44)	ns
L2 colonic	11 (23)	17 (17)	ns
L3 ileocolonic	13 (27)	38 (39)	ns
L4 isolated upper disease^b^	none	none	
CD behavior			
B1 non-stricturing, non-penetrating	47 (98)	81 (83)	ns
B2 stricturing	19 (40)	39 (40)	ns
B3 penetrating	13 (27)	17 (17)	ns
P perianal disease modifier	5 (10)	5 (5)	
**Medication at inclusion** n (%)			
Aminosalicylic acid (5-ASA)	82 (59)	69 (42)	<0.001(χ^2^)
Azathioprine (AZA)/Methotrexate (MTX)	27 (19)	29 (18)	ns
Biological^c^	18 (13)	48 (29)	<0.001(χ^2^)
Prednisolone^d^	18 (13)	23 (14)	ns

Explanations: For one CD patient in CF, information was lacking on disease localization, and for nine CD patients on behavior classification

*Abbreviation: ns* non-significant

^a^ IBD classification as provided by the Montreal classification [17]

^b^L4 is added to L1-L3 when concomitant upper gastrointestinal disease is present

^c^Biological = tumor necrosis factor alpha and anti-integrin and other biological medication

^d^Use of prednisone during the last year before study enrolment

Before we compared the NF and the CF model concerning HRQoL, we adjusted statistically significant differences in sociodemographic and clinical characteristics by weighting relevant variables as follow: age, age categories, disease duration, Aminosalicylic acid (5-ASA) and Biologics.

### Health-related quality of life

A total of 303 participants completed the IBDQ questionnaire. Comparing the NF model with the CF model, we found statistically significant differences in median score, in both IBDQ total score and dimensional scores, as presented in Table 2.

**Table 2:** Health-related quality of life assessment (IBDQ).  Patient-reported outcome score in the NF and CF models, *n* = 303^a^

Follow-up model	Nurse-led Median	Conventional Median	*P-value*
**Total IBDQ** (*n* = 139/164)	184.0	171.0	< .001
**Bowel function I** (*n* = 139/164)	44.0	41.0	0.02
**Bowel function II** (*n* = 139/164)	26.0	25.0	0.02
**Emotional function I** (*n* = 139/164)	56.0	53.0	< 0.01
**Emotional function II** (*n* = 139/164)	31.0	30.0	0.03
**Social function** (*n* = 139/164)	26.0	24.0	< .001

^a^There was one patient missing in the NF group because of an incomplete version of the IBDQ

A Mann-Whitney U test was performed to compare NF versus CF

In one of the intervention centers, the IBD nurse at the outpatient clinic did not regularly follow-up patients on biologics, because this was taken care of by IBD nurses at the infusion ward, with supervision by a physician. We therefore conducted the same test after excluding this center, with the same statistical results concerning HRQoL.

### IBDQ and symptom severity categories

The IBDQ total score was divided into three different symptom categories as presented in Table 3 [24].

**Table 3:** IBDQ scores divided into severity categories

	Nurse-led total: 139 n (%)	Conventional total: 164 n (%)
Severe active disease: IBDQ ≤ 130	9 (7)	31 (19)
Moderate active disease: IBDQ > 130 < 180	53 (38)	65 (40)
Remission: IBDQ ≥ 180	77 (55)	68 (41)

A chi-square test of independence was performed to compare the NF model with the CF model with regard to their association with IBDQ disease severity categories. The relation between these variables was statistically significant (*p* = 0.002) with a higher proportion of patients classified as in remission in the NF population.

Additionally, patients in the NF group had a statistically significant higher frequency of consultations over a two-year period, than those in the CF group (*p* < .000) with a mean of 3.0 (SD: 1.5) and 2.2 (SD: 2.1), respectively.

## Discussion

In IBD follow-up care, medical treatment has always been, and still is, most essential to control the clinical symptoms. These symptoms may affect the HRQoL and potentially lead to absence from work and school, affect family and social life as well as affect the patient’s mental wellbeing to a significant degree.

In this cross-sectional study, we observed that IBD patients attending nurse-led follow-up reported comparable HRQoL outcomes to conventional follow-up. Additionally, the same population had more frequent consultations.

Despite a trend of higher IBDQ score in all domains in the NF model, the overall result in our study did not reach the limit of 16 points, defined as clinically significant. Based on these findings, we conclude the IBD nurse-led follow-up to be non-inferior to the conventional model concerning HRQoL. It might be speculated if our observation concerning a throughout trend of increased HRQoL scores in IBD patients attending NF, might be related to the more extensive consultation time giving the opportunity to focus on issues such as coping and empowerment. Indeed, prior recommendations have highlighted the importance of psychological support, which helps the patients to cope with aspects of living with a chronic disease and hence increases HRQoL [15, 25]. Even so, neither Nightingale et al. who evaluated the effect of an IBD nursing service [26], nor Jelsness-Jørgensen et al. who compared a nurse-led and a conventional follow-up approach were able detect any changes in HRQoL [13].

However, a notable outcome concerning HRQoL is a clinical significance improvement in the score of the social domain, underlining the advantage of the model with patient-centred approach including psychosocial support. This is in line with the N-ECCO consensus, which emphasizes the IBD nurse's key role in providing effective, comprehensive and accessible care [12].

Seeing the patients over a long period of time is one advantage that nurses potentially have compared to other groups of healthcare professionals, enabling coordination and communication [12]. Indeed Fiorini et al., summarized the IBD nurse role as important in the management of IBD including patients counseling, education, physical and emotional support, as well as being accessible for patients by phone or email [27]. In clinical settings many patients have appreciated the opportunity to call an IBD nurse as needed [25]. This is in great accordance to our experience, where accessible care constitutes security for the patients.

We observed that patients in the NF group had less severe disease than those in the CF group. Taken together with a higher number of patients on biologics in the CF group, these differences may potentially reflect a systematic selection bias. Moreover, a larger proportion of patients attending CF were diagnosed with CD, of younger age and had a shorter disease duration than the NF group. Of course, these are all factors that may contribute to the observed differences in HRQoL between the NF and CF group. For instance, CD is a disease typically associated with lower HRQoL compared to UC patients [21] and longer disease duration has also been associated with improved HRQoL [28].

Despite the lack of studies investigating the role of and follow-up provided by IBD nurses, some authors have demonstrated that nurse-led follow-up result in fewer hospital admissions, shorter time from relapse to intervention, increased patient satisfaction and cost reductions [13, 26, 29–31]. In the current study we observed an elevated number of consultations in the NF versus CF group. Noteworthy, one-third of the patients in the CF population did not have regular outpatient checkups by a physician over a two-year period. Even though we do not have exact data to explain the background of this observation, it could reflect limited access to gastroenterologists in combination with often long distances to hospitals, which indeed is the case particularly in Northern Norway. The latter may also underscore the need for alternative forms of access to follow-up care, such as telephone and virtual consultations. As in our study, Jelsness-Jørgensen et al. also observed a higher number of consultations among patients attending NF. Interestingly the same authors found that the time from relapse to start of treatment was statistic significantly shorter in the NF model [13]. This finding was attributed to the skill of the IBD nurse and the regularity of the outpatient visits in the NF model [13].

IBD care provided by a multidisciplinary team has been stated as the ideally follow-up model [32]. This model of care includes core members like gastroenterologists, surgeons, radiologists, an IBD nurse specialist and stoma nurse [10]. This model of care may not be possible to organize at small units. In this case the MT model can be a good alternative with an IBD nurse in a central role in the daily follow-up management.

The study has some limitations. The cross-sectional design makes it difficult to draw conclusions about the impact of the NF model on HRQoL. In addition, the result may be influenced by the response rate (47%) and the fact that we do not know who responded. This may represent a selection bias and a higher number of intervention centers would probably have given a more robust result. However, the actual number reflect the situation with very few IBD nurse led outpatient clinics when the study was planned.

In one of NF-centers most of the patients treated with biologics regularly consulted IBD nurses in the infusion ward, and were consequently not included in the NF population. This can be the main explanation for why we detected a lower number of patients treated with biologics in the NF group. This finding can indicate a selection bias concerning one of the intervention centers and could have influenced the IBDQ scores. On the other hand, when we excluded this center from the HRQoL analysis, the result remained unchanged.

A strength of the study was that all included participants attended national public hospitals, with a potential of equal standards of care, thus forming a basis for comparison.

Further research in these areas is necessary, and this must be multidimensional, by assessing both clinical endpoints, the patient's own experiences and overall cost analyses.

## Conclusion

Nurse-led models are not inferior to conventional models with regards to patient reported HRQoL. Further studies are needed to advance efforts to implement these models and increase access to IBD care.

## Data Availability

All data generated or analysed during this study are included in this published article.

## Declarations

The study was approved by the Norwegian Data Protection Officer (reference number: 44439) and in accordance with the Personal Data Regulations cf. § 7–27 (https://lovdata.no/dokument/SFO/forskrift/2000-12-15-1265/KAPITTEL_7-4#%C2%A77-27).

After application and assessment by the Norwegian Research Ethics Committees for Medical and Health Research Ethics (REK Nord) (https://rekportalen.no/#hjem/home), this project was not subject to notification according to national regulations cf. Helseforskningslovens § 10 (https://lovdata.no/dokument/NL/lov/2008-06-20-44) and cf. Forskningsetikkloven § 4 (https://lovdata.no/dokument/NL/lov/2017-04-28-23). Reference number: 2013/1397/REK Nord.

Each participant was explained the research aims and asked to sign an informed consent before completing the study surveys. Subjects were allowed to drop out at any time.

## References

[1] Burisch J, Jess T, Martinato M, Lakatos PL, ECCO-EpiCom (2013). The burden of inflammatory bowel disease in Europe. J Crohn's Colitis.

[2] Irvine E (1996). Quality of life in inflammatory bowel disease and other chronic diseases. Scand J Gastroenterol.

[3] Loftus EV (2004). Clinical epidemiology of inflammatory bowel disease: incidence, prevalence, and environmental influences. Gastroenterology.

[4] Verhoef MJ, Sutherland LR (1990). Outpatient health care utilization of patients with inflammatory bowel disease. Dig Dis Sci.

[5] Berntsen G, Høyem A, Gammon D. The health service seen from a patient perspective.[Prosjektrapport-Helsetjenesten sett fra pasientens ståsted. Pasientforløp ved langvarige og komplekse behov i Troms-og Ofoten]. Report Norway: Norwegian center for integrated care and telemedicine, Helse Nord RHF, Norwegian center for integrated care and telemedicine, Helse Nord RHF. 2014.

[6] Epstein RM, Street RL. The values and value of patient-centered care. Ann Fam Med. 2011;9(2):100–3. 10.1370/afm.1239 PMC3056855 21403134

[7] Jelsness-Jørgensen L-P, Bernklev T, Hovde Ø, Prytz Berset I, Huppertz-Hauss G, Moum B (2016). Patients’ perceptions of quality of care and follow-up in inflammatory bowel disease. Scand J Gastroenterol.

[8] Irvine E (2004). Patients' fears and unmet needs in inflammatory bowel disease. Aliment Pharmacol Ther.

[9] Morar P, Read J, Arora S, Hart A, Warusavitarne J, Green J, et al. Defining the optimal design of the inflammatory bowel disease multidisciplinary team: results from a multicentre qualitative expert-based study. Front Gastroenterol. 2015:flgastro-2014-100549. 10.1136/flgastro-2014-100549 PMC5369592 28839825

[10] Calvet X, Panés PJ, Alfaro N, Hinojosa J, Sicilia B, Gallego M (2014). Delphi consensus statement: quality indicators for inflammatory bowel disease comprehensive care units. J Crohn's Colitis.

[11] Krogstad U, Hofoss D, Veenstra M, Hjortdahl P (2006). Predictors of job satisfaction among doctors, nurses and auxiliaries in Norwegian hospitals: relevance for micro unit culture. Hum Resour Health.

[12] Kemp K, Dibley L, Chauhan U, Greveson K, Jaghult S, Ashton K (2018). Second N-ECCO Consensus Statements on the European Nursing Roles in Caring for Patients with Crohn's Disease or Ulcerative Colitis. J Crohns Colitis.

[13] Jelsness-Jørgensen L-P, Bernklev T, Henriksen M, Torp R, Moum B (2012). Is patient reported outcome (PRO) affected by different follow-up regimens in inflammatory bowel disease (IBD)? A one year prospective, longitudinal comparison of nurse-led versus conventional follow-up. J Crohn's Colitis.

[14] Dibley L, Bager P, Czuber-Dochan W, Farrell D, Jelsness-Jørgensen L-P, Kemp K (2017). Identification of research priorities for inflammatory bowel disease nursing in Europe: a Nurses-European Crohn’s and colitis organisation delphi survey. J Crohn's Colitis.

[15] Elkjaer M, Moser G, Reinisch W, Durovicova D, Lukas M, Vucelic B (2008). IBD patients need in health quality of care ECCO consensus. J Crohn's Colitis.

[16] World Health Organization. International statistical classification of diseases and related health problems (11th ed.). 2022 [updated May 2019 (WHA72.15); cited 2022 November 15th]. Available from: https://www.who.int/standards/classifications/classification-of-diseases.

[17] Satsangi J, Silverberg M, Vermeire S, Colombel J (2006). The Montreal classification of inflammatory bowel disease: controversies, consensus, and implications. Gut.

[18] Guyatt G, Mitchell A, Irvine EJ, Singer J, Williams N, Goodacre R (1989). A new measure of health status for clinical trials in inflammatory bowel disease. Gastroenterology.

[19] Irvine EJ (1999). Development and subsequent refinement of the inflammatory bowel disease questionnaire: a quality-of-life instrument for adult patients with inflammatory bowel disease. J Pediatr Gastroenterol Nutr.

[20] Chen X-L, Zhong L-h, Wen Y, Liu T-W, Li X-Y, Hou Z-K (2017). Inflammatory bowel disease-specific health-related quality of life instruments: a systematic review of measurement properties. Health Qual Life Outcomes.

[21] Bernklev T, Jahnsen J, Aadland E, Sauar J, Schulz T, Lygren I (2004). Health-related quality of life in patients with inflammatory bowel disease five years after the initial diagnosis. Scand J Gastroenterol.

[22] Bernklev T, Moum B, Moum T (2002). Quality of life in patients with inflammatory bowel disease: translation, data quality, scaling assumptions, validity, reliability and sensitivity to change of the Norwegian version of IBDQ. Scand J Gastroenterol.

[23] Irvine E (1993). Quality of Life–rationale and methods for developing a disease-specific instrument for inflammatory bowel disease. Scand J Gastroenterol.

[24] Gregor JC, McDonald JW, Klar N, Wall R, Atkinson K, Lamba B (1997). An evaluation of utility measurement in Crohn's disease. Inflamm Bowel Dis.

[25] Schoultz M, Macaden L, Watson AJ (2016). Co-designing inflammatory bowel disease (Ibd) services in Scotland: findings from a nationwide survey. BMC Health Serv Res.

[26] Nightingale AJ, Middleton W, Middleton SJ, Hunter JO (2000). Evaluation of the effectiveness of a specialist nurse in the management of inflammatory bowel disease (IBD). Eur J Gastroenterol Hepatol.

[27] Fiorino G, Allocca M, Chaparro M, Coenen S, Fidalgo C, Younge L (2018). ‘Quality of Care’ Standards in Inflammatory Bowel Disease: A Systematic Review. J Crohn's Colitis.

[28] Jäghult S, Saboonchi F, Johansson U-B, Wredling R, Kapraali M (2011). Identifying predictors of low health-related quality of life among patients with inflammatory bowel disease: comparison between Crohn’s disease and ulcerative colitis with disease duration. J Clin Nurs.

[29] Leach P, De Silva M, Mountifield R, Edwards S, Chitti L, Fraser RJ (2014). The effect of an inflammatory bowel disease nurse position on service delivery. J Crohn's Colitis.

[30] Sack C, Phan V, Grafton R, Holtmann G, van Langenberg D, Brett K (2011). A chronic care model significantly decreases costs and healthcare utilisation in patients with inflammatory bowel disease. J Crohn's Colitis.

[31] Coenen S, Weyts E, Vermeire S, Ferrante M, Noman M, Ballet V (2017). Effects of introduction of an inflammatory bowel disease nurse position on the quality of delivered care. Eur J Gastroenterol Hepatol.

[32] Panés J, O'Connor M, Peyrin-Biroulet L, Irving P, Petersson J, Colombel J-F (2014). Improving quality of care in inflammatory bowel disease: what changes can be made today?. J Crohn's Colitis.

